# Early post‐induction augmented therapy improves outcome in children and adolescents with T‐cell acute lymphoblastic leukemia

**DOI:** 10.1002/cnr2.1703

**Published:** 2022-08-12

**Authors:** Mayada Abu Shanap, Haytham Al Jabour, Rawad Rihani, Hasan Hashem, Amal Abu Ghosh, Abdelghani Tbakhi, Nazmi Kamal, Iyad Sultan, Faris Madanat

**Affiliations:** ^1^ Department of Pediatric King Hussein Cancer Center Amman Jordan; ^2^ Department of Cell Therapy & Applied Genomics King Hussein Cancer Center Amman Jordan; ^3^ Department of Pathology and Laboratory medicine King Hussein Cancer Center Amman Jordan

**Keywords:** children, cranial radiation, intensification therapy, relapse, T‐cell acute leukemia

## Abstract

**Introduction:**

T‐cell acute lymphoblastic leukemia (T‐ALL) accounts for approximately 15% of all newly diagnosed ALL in children and adolescents and is associated with worse outcomes compared to pre‐B ALL. We aimed to decrease T‐ALL relapses by intensifying our regimen.

**Methods:**

Patients with T‐ALL were treated using two different regimens; before September 2014, patients were treated per St. Jude Total XV protocol; subsequently, a major change was adopted by adding two intensive blocks: FLAG and Reintensification. Cranial radiation was limited to patients with WBC ≥ 100 k/μl at diagnosis and/or patients with CNS2/CNS3 status.

**Results:**

Between June 2005 and April 2020, a total of 100 patients (76 males) were treated and followed up for a median of 70 months (range 14–181). Median age at diagnosis was 9 years (range 0.5–17.8). Forty‐eight patients were diagnosed after September 2014 and received the augmented regimen; their median follow up was 46 months (range 14–74). The 5‐year‐EFS estimates for patients who received the augmented regimen versus standard regimen were 87% ± 4.9% versus 67% ± 6.8% (*p* = .03); and the 5‐year‐OS estimates were 87% ± 5.1% versus 71% ± 6.3% (*p* = .06), respectively. Treatment related mortality (TRM) was reported in two patients treated per standard regimen but none for patients who received the augmented regimen.

**Conclusions:**

We implemented a novel approach with early intensification added to a backbone of modified St. Jude Total‐XV regimen for patients with T‐ALL that resulted in improved outcome with no treatment related mortality.

## 1 INTRODUCTION

T‐cell acute lymphoblastic leukemia (T‐ALL) represents about 15% of newly diagnosed cases of childhood acute lymphoblastic leukemia (ALL). Historically, T‐ALL has had lower event‐free survival (EFS) and overall survival (OS) compared with precursor B‐cell ALL (B‐ALL). Patients with T‐ALL often have adverse clinical characteristics at diagnosis such as: older age, a markedly high white blood cell count (Wbc), and central nervous system involvement. Additionally, T‐ALL patients tend to have suboptimal response at end of induction defined by failure of induction or persistent MRD.^1^, ^2^, ^3^, ^4^

Frontline intensive therapy with a four‐drug induction containing: dexamethasone, anthracycline, vincristine and asparaginase followed by augmented BFM‐like consolidation has resulted in improved survival in T‐cell ALL. However, early relapse remains the leading cause of failure of therapy and death.^5^, ^6^, ^7^, ^8^, ^9^, ^10^, ^11^, ^12^, ^13^

The outcome of children and adolescents with T‐ALL treated at our center per modified St. Jude total XV protocol was reported in 2014; the 5‐year‐OS and EFS of Early T‐cell precursor acute lymphoblastic leukemia (ETP) were 79.6% and 77.1%; while for non‐ETP were 78.5% and 65.2%, respectively.^14^

We hypothesized that early and intensive therapy could improve outcomes in T‐cell ALL. Subsequently, we changed our regimen to include fludarabine, high‐dose cytarabine, and etoposide as part of an early intensive post‐induction chemotherapy treatment. The use of enhanced therapy in European protocols for high‐risk leukemia prompted this approach.^7^, ^15^

We report here our results using both regimens (standard and augmented) in children and adolescents who presented to our center with untreated T‐ALL over a period of over 16 years.

## 2 METHODS

### 2.1 Patients

After obtaining approval of our institutional review board (IRB #:19KHCC34), we retrospectively reviewed all patients who presented with T‐ALL to our department from January 2005 until April 2020. Patients' records were identified by querying our departmental database. We included all patients with T‐ALL who presented at our institution and treated per modified St Jude Total XV protocol. Patients who presented with relapsed disease, received previous therapy before referral and patients who underwent hematopoietic cell transplantation (HCT) in first remission were excluded.

### 2.2 Treatment

#### 2.2.1 Standard protocol

**FIGURE 1:**
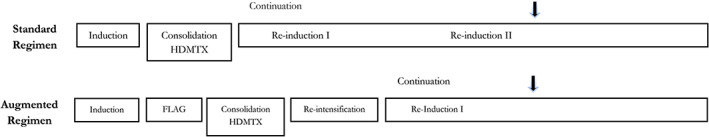
Treatment plan of T‐cell acute lymphoblastic leukemia. 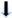 Cranial Radiation

All T‐ALL patients were treated per modified St. Jude total XV protocol. Treatment consisted of remission induction, consolidation, and continuation (Figure 1). Remission induction composed of dexamethasone, vincristine, daunorubicin, and asparaginase. Subsequent induction therapy consisted of cyclophosphamide, mercaptopurine, and cytarabine (Table S1A). On hematopoietic recovery (between days 43 and 46), the minimal residual disease was assessed, and consolidation therapy was begun with four doses of high‐dose methotrexate (HD‐MTX) at 5 g/m^2^ and daily mercaptopurine (Table S1C). During initial continuation therapy, patients received an intensive schedule of asparaginase chemotherapy, daily mercaptopurine interrupted with pulses of doxorubicin, vincristine, and dexamethasone in addition to two reinduction cycles, followed by three rotating drug pairs. Continuation treatment lasted 120 weeks in girls and 146 weeks in boys (Table S1E).

#### 2.2.2 Augmented regimen

In September 2014, we modified our regimen for T‐ALL by adding two blocks of chemotherapy to all patients: FLAG and reintensification (Figure 1). FLAG as (fludarabine 15 mg/m^2^ × 4 days, high‐dose cytarabine as 2 g/m^2^ × 4 days) in patients with negative MRD or MRD ≥ 0.01% and <1%, and FLAG as (fludarabine 30 mg/m^2^ × 4, high‐dose cytarabine as 2 g/m^2^ × 4) if MRD ≥ 1% (Table S1B). Reintensification (high‐dose cytarabine, dexamethasone, etoposide, peg‐asparaginase) was given to all patients (Table S1D); during continuation: Reinduction I was given and reinduction II was omitted (Table S1E).

#### 2.2.3 CNS‐directed therapy

All patients received triple intrathecal therapy (IT) on days 1 and 15 of induction with additional doses on days 8 and 22 in patients with peripheral Wbc ≥ 50 000/μl at presentation and continued through the consolidation and continuation therapy (total number 21–23). Cranial radiation (CRT) was given to patients at increased risk of CNS relapse at continuation week 21, including patients with Wbc count ≥ 100 000/μl at presentation (CRT dose of 12Gy) and patients with CNS‐2 or CNS‐3 status at diagnosis or those who had a traumatic lumbar puncture with blasts (CRT dose of 18Gy).

### 2.3 Definitions and statistical analysis

According to our clinical practice guidelines which were drafted in 2005, failure of induction is defined as MRD ≥ 1% at day 35–42 of remission induction. Refractory disease (RD) was defined as leukemia persistence in patients surviving induction. Relapse was defined as disease recurrence at any site after achieving remission. Treatment‐related mortality (TRM) was defined as death in remission in patients receiving treatment for ALL. Rapid early responder (RER) was defined as M1 marrow (<5% blasts) on day 15, and with negative MRD status (<0.01%) at the end of Induction. Slow early responder (SER) was defined as M2 (5%–25% blasts) or M3 (>25% blasts) marrow on Day 15 or positive MRD status (≥0.01%) at end of induction.

OS was measured from the time of entry in the protocol to the time of death by any cause or last follow‐up. Event‐free survival (EFS) was defined from the time of entry in the protocol to the date of relapse or date of refractory disease post FLAG and/or reintensification chemotherapy, date of second cancer or death from any cause or date of last follow up. Data analysis was performed using R software (V4.0.4.) with calculation of Kaplan–Meier estimates and log‐rank tests using “survival” package and drawing survival curves using “survminer” package. We iterated through all possible covariates to calculate the *p*‐values using log‐rank test. Significant covariates were included in a multivariate Cox‐regression model. Chi‐square test and Mann–Whitney test were used to evaluate categorical and numeric variables, respectively. A *p*‐value of ≤.05 was considered significant.

## 3 RESULTS

### 3.1 Patient's characteristics

One hundred patients were included in this study; median age at diagnosis was 9.2‐years (range 0.5–17.8 year); 76% were males; 30 patients (30%) presented with initial WBC count ≥200 × 10^9^/L; the majority of the patients (76%) had high‐risk disease per NCI classification. CNS involvement was documented in 15 patients (15%). There were no significant differences in the distributions of age (*p* = .84), gender (*p* = .52), Wbc (*p* = .06), CNS status at diagnosis (*p* = .87), and NCI risk (*p* = .36) between the two groups (Table 1).

**TABLE 1:** Characteristics of patients at diagnosis treated on standard versus augmented therapy and outcomes

	Whole group (*N* = 100)	Standard therapy (*N* = 52)	Augmented therapy (*N* = 48)	*p* Value
Age (years)(Median (range))	9.2 years(0.5–17.8)	8.9 years(0.5–17.8)	9.7 years(2.1–17.6)	.4
*Age*				
<1 or ≥10 year	48	24(46%)	24(50%)	.836
≥1 and <10 year	52	28(54%)	24(50%)	
*Gender*				
Male	76	37(71%)	39(81%)	.516
Female	24	15(29%)	9(19%)	
*WBC at diagnosis*				
<100	54	24(46%)	30(63%)	.064
≥100	46	28(54%)	18(37%)	
*CNS status*				
1	85	43(83%)	42(88%)	
2	8	5(9%)	3(6%)	.878
3	7	4(8%)	3(6%)	
*NCI risk*				
SR	24	10(19%)	14(29%)	.361
HR	76	42(81%)	34(71%	
*Bone marrow at day 15*				
M1	73	40(77%)	33(69%)	
M2	18	9(17%)	9(19%)	.129
M3	9	3(6%)	6(13%)	
*Bone marrow at end of Induction*				
M1	94	50(96%)	44(92%)	
M2	5	2(4%)	3(6%)	.365
M3	1	0	1(2%)	
*MRD at end of induction*				
<0.01%	85	48(92%)	37(77%)	
≥0.01%	15	4(8%)	11(23%)	.004
*Early response to treatment*				
Rapid early responder (RER)	68	38(73%)	30(63%)	.046
Slow early responder (SER)	32	14(27%)	18(37%)	
*Cranial XRT*				
Yes	21	11(21%)	10(21%)	.100
No	79	41(79%)	38(79%)	
*Status at last follow up*				
Alive in CCR	78	36(69%)	42(88%)	.04
Dead/cause	22	16(31%)	6(12%)	
Relapse		14	2	
TRM		2	–	
Second cancer		–	1	
Refractory		–	3	

### 3.2 Response to induction therapy

Thirty‐eight (73%) patients had rapid early response in standard group versus 30 (63%) patients in augmented therapy group (*p* = .046). MRD evaluation at end of induction showed ≥0.01% involvement in 8% and 23% (*p* = .004) of patients in standard and augmented groups, respectively.

### 3.3 Outcomes and prognostic factors

**FIGURE 2:**
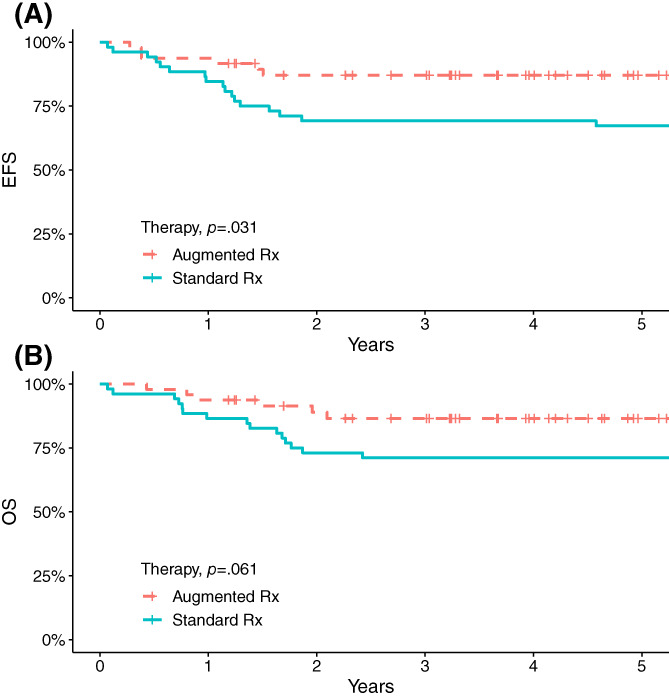
Event‐free survival (EFS) (A) and overall survival (OS) (B) of patients with T‐acute lymphoblastic leukemia treated per standard and augmented therapy groups

At a median follow up of 70 months (range 14–181), the 5‐year EFS was 76% ± 4.4% and the 5‐year OS was 78% ± 4.2% for the whole group. When compared using log‐rank test, the 5‐year EFS for patients treated in the augmented therapy group was 87% ± 4.9% which is significantly better than the standard therapy group estimate of 67% ± 6.5% (*p* = .03) (Figure 2A). However, there was no statistically significant difference in 5‐year OS between the 2 groups 87% ± 5.1% in augmented versus 71% ±6.2% in standard group (*p* = .061) (Figure 2B).

In a univariate analysis model, other factors associated with better EFS and OS were: Response to therapy at day 15 of remission induction (*p* = .024 and *p* = .02, respectively) and cranial radiation (*p* = .00001 and *p* = .01, respectively) (Table S2).

**FIGURE 3:**
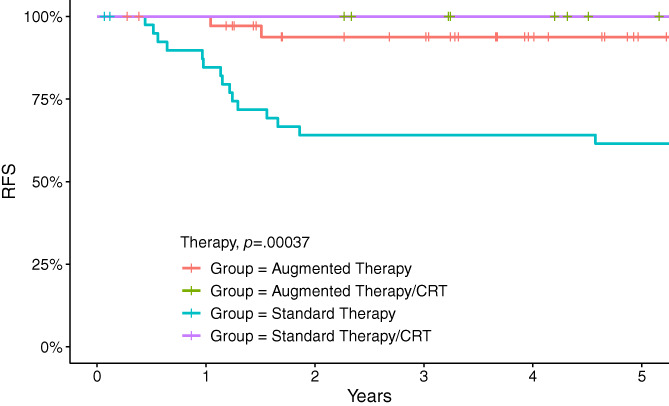
Relapse free survival (RFS) in patients with T‐acute lymphoblastic leukemia treated in standard and augmented therapy with or without cranial radiation (CRT)

We opted to study the effect of cranial radiation on the relapse as outcome in both groups using log rank test. Notably, none of the patients (*N* = 21) who received CRT relapsed in either group; the 5‐year relapse free survival (RFS) in patients who received CRT in both groups was 100% ± 0%, and in patients who did not receive CRT in augmented and standard therapy group was 94% ± 4.2% and 62% ± 7.7% (*p* = .0004), respectively (Figure 3).

In total, 79 patients did not received CRT (*N* = 41 in standard therapy, *N* = 38 in augmented therapy). Seventeen (22%) of 79 patients who did not receive CRT had bone marrow or isolated extramedullary relapse. Among these 17 patients, 15/79 (19%) were treated in standard therapy group and 2/79 (3%) in the augmented therapy group (Table 2).

**TABLE 2:** Relapse in standard and augmented therapy group based on cranial radiation therapy

	Relapse in standard therapy group	Relapse augmented therapy group
Group I/received CRT (*N* = 21)	–	–
Group II/did not receive CRT (*N* = 79)	15/79(19%)	2/79(3%)
Did not receive CRT per protocol (*N* = 48)^a^	7/48 (15%)	0/48(0%)
Did not receive for other reasons (*N* = 31)^b^	8/31 (26%)	2/31(6%)

^a^ CRT was not indicated per protocol.

^b^ CRT was indicated; not given due to parent's refusal.

### 3.4 Toxicity

Regarding hematological toxicities all patients in the augmented therapy group were admitted with febrile neutropenia post each block of intensive therapy, seven of 48 patients needed intensive care and another four patients had invasive fungal infections, all are alive. Notably, all had grade 3 anemia, and grade 3–4 thrombocytopenia; the toxic effects of augmented therapy were considerable but manageable with no treatment related death. Nonhematological toxicities between two groups were comparable.

### 3.5 Events

There were 17 events in the standard therapy group (15 relapses, and 2 treatment‐related death due to liver failure and sepsis), while patients treated in the augmented therapy group had 6 events (one died of second cancer/AML, two relapsed, and three had refractory disease). The median time to relapse for all patients was 13.8 months (range 6–55). Eleven of 17 patients who relapsed (65%) had isolated BM relapse, four had isolated extramedullary relapse, and two had combined relapse (BM + CNS). Six patients (35%) achieved CR2 after salvage chemotherapy, of them three patients underwent allogenic‐HCT in CR2, only one patient is alive in continuous complete remission (CCR) post‐transplant; all others (*N* = 5) succumbed due to second relapses.

## 4 DISCUSSION

In this retrospective study, we reviewed the outcome of two consecutive protocols for T‐cell ALL treated at our center over 16 years. We opted to add additional intensive therapy to the St. Jude Total XV protocol that had been used with less optimal outcomes.^14^ We adopted two intensification blocks using fludarabine, high‐dose cytarabine and G‐CSF for the FLAG regimen and high‐dose cytarabine, etoposide, dexamethasone and peg‐asparaginase for the second intensification cycle; the rationale of this change is early administration of high‐dose chemotherapy. These combinations were previously reported to be effective in high risk and refractory relapsed ALL.^15^, ^16^, ^17^ Using this augmented regimen, there was a significant improvement in the EFS at 87% compared to 67% for patients treated with the standard therapy. This improvement in EFS was seen irrespective of the early response at day 15 and end of induction.

Attempts to intensify treatment of patients with T‐ALL are well‐reported in the literature^7^, ^8^, ^10^, ^18^, ^19^, ^20^; we adopted unique novel upfront intensive therapy which results in outcome in line with other contemporary clinical trials of T‐ALL with no treatment related mortality.

The observation of a high rate of positive MRD in the augmented group at end of induction, in comparison to the historical standard group; though both received the same induction regimen, was not due to either biological or clinical differences between both groups but rather due to implementation of newer flowcytometry techniques for MRD detection after 2014 thus minimizing false‐negative MRD reporting.

Additionally, we have found that intensification of therapy overcame the adverse effect of positive MRD at end of induction. Notably, 11 patients in the augmented group had positive MRD (≥0.01%) and/or M2, M3 marrow at the end of induction. Eight of the 11 patients (73%) achieved remission with negative MRD (<0.01%) post FLAG chemotherapy, 7 of the 8 patients (88%) remained in CCR at date of last follow‐up.

Currently, the use of cranial radiation (CRT) for pediatric patients with T‐ALL is variable,^21^ with some cooperative groups administer CRT to all T‐cell patients,^22^ while others adopt a risk‐directed CRT approach.^23^, ^24^ Some groups reserve CRT for patients with CNS involvement^19^, ^25^, ^26^ while others omitted CRT for all patients treated on St. Jude total XV and XVI, EORTC/58881, DCOG‐ALL 9.^6^, ^11^, ^15^, ^27^

In our cohort, cranial radiation contributed to a significant improvement in EFS (*p* = .00001) and OS (*p* = .01) in both groups. We compared the outcomes per CRT, the 5‐year relapse free survival (RFS) in patients who received CRT in both groups was 100% ± 0%. The RFS in patients who did not receive CRT in the augmented and standard therapy groups was 94% ± 4.2% and 62% ± 7.7% (*p* = .0004), respectively. This indicates that effective systemic CNS‐directed chemotherapy in the augmented therapy group may compensate for the lack of CRT. This significant finding may change our current practice of risk‐directed CRT to omitting CRT in all patients.

Our study has multiple limitations that adds to its retrospective design. First, the small number of patients in each cohort and the small number of events limit the power of our statistical analysis. Second, patients treated per this augmented therapy need a longer duration of follow up to determine the durability of this excellent cure rate. Third, we are comparing two groups treated in different periods; the MRD detection methods have greatly improved at our institution over the past 10 years. Additionally, the ability to provide supportive care has drastically improved. Fourth, after 2014 all patients with T‐ALL received two blocks of intensive chemotherapy regardless of the MRD response after remission induction; including those with rapid early response, thus, low‐risk T‐cell ALL might be over treated. The toxic effects of the augmented therapy were considerable but manageable. Hematological toxicities were balanced against the absence of treatment‐related mortality due to enhanced supportive care at our center.

In summary, we showed that early intensification of systemic and CNS‐directed chemotherapy results in markedly improved outcome in T‐ALL patients as it significantly decreases the risk of relapse. In the future, response‐based de‐escalation of therapy and Escalation of therapy based on MRD is highly needed as this could limit unnecessary intensive therapy and reduce treatment‐related mortality and morbidity. The use of prophylactic cranial radiation therapy in the treatment of patients with T‐ALL is declining given the higher rates of neurocognitive sequelae, endocrinopathies, and secondary malignancies associated with CRT. We found that RFS in patients who did not receive CRT was significantly lower in patients who were treated with our augmented therapy compared to standard therapy; this was likely related to effective intensive systemic CNS directed therapy. Omitting CRT in setting of augmented therapy is warranted.

## AUTHOR CONTRIBUTIONS

**Mayada Abu Shanap:** Conceptualization (lead); data curation (lead); formal analysis (equal); methodology (lead); project administration (lead); resources (lead); software (supporting); supervision (lead); validation (equal); visualization (equal); writing – original draft (lead); writing – review and editing (lead). **Haytham Aljbour:** Data curation (equal). **Rawad Rihani:** Conceptualization (equal). **Hasan Hashem:** Conceptualization (equal). **Amal Abu Ghosh:** Conceptualization (equal). **Abdelghani Tbakhi:** Conceptualization (equal). **Nazmi Kamal:** Conceptualization (equal). **Iyad Sultan:** Data curation (equal); formal analysis (equal); software (equal); validation (equal). **Faris Madanat:** Conceptualization (equal); validation (equal); visualization (equal).

## CONFLICT OF INTEREST

The authors declare that there is no conflict of interest.

## ETHICS STATEMENT

This study was approved by our institutional review board (IRB #:19KHCC34) at King Hussein Cancer Center, Jordan. The research teamadhered to the ethical principles of the Helsinki. The study is retrospective; so, the requirement of informed consent was waived by institutional reviewboard.

## Supporting information


**Appendix S1** Supporting Information

## Data Availability

Primary data will be made available from the corresponding authors upon request to protect patient privacy. Data availability may be subject to approval from King Hussein Cancer Center‐IRB.
