# Supplementary material for: Early post‐induction augmented therapy improves outcome in children and adolescents with T‐cell acute lymphoblastic leukemia
Source: Cancer Rep (Hoboken). 2022 Aug 12;6(2):e1703. doi: 10.1002/cnr2.1703 (PMC9940001; doi:10.1002/cnr2.1703)
Supplement: Supplementary file 1 — Appendix S1 Supporting Information [file CNR2-6-e1703-s001.docx]

**Supplementary Appendix**

**Table 1. Modified total VX protocol in standard therapy and augmented therapy group**

**A. Remission Induction**

|  | **Standard Therapy** | | **Augmented Therapy** | |
| --- | --- | --- | --- | --- |
| **Agent** | **Dose** | **Schedule** | **Dose** | **Schedule** |
| Dexamethasone | 8 mg/m2 | Days 1-28 | 8 mg/m2 | Days 1-28 |
| Vincristine | 1.5 mg/m2 per week | Days 1, 8, 15, 22 | 1.5 mg/m2 per week | Days 1, 8, 15, 22 |
| Daunorubicin | 25 mg/m2 per week | Days 1, 8 | 25 mg/m2 per week | Days 1, 8 |
| L-Asparaginase or #  PEG-asparaginase | 10,000 U/m2  IM (thrice weekly)  2,500 U/m2 IM | Days 6, 8, 10, 12, 14, 16, (19, 21, 23)  Days 3, (16) | 10,000 U/m2  IM (thrice weekly)  2,500 U/m2 IM | Days 6, 8, 10, 12, 14, 16, (19, 21, 23)  Days 3, (16) |
| Cyclophosphamide | 1000 mg/m2 IV | Day 22 | 1000 mg/m2 IV | Day 22 |
| Cytarabine | 75 mg/m2 IV | Days 23-26, 30-33 | 75 mg/m2 IV | Days 23-26, 30-33 |
| Mercaptopurine | 60 mg/m2/dose orally | Days 22-35 | 60 mg/m2/dose orally | Days 22-35 |
| Triple Intrathecal (IT) | Age dependent | Days 1, (8), 15, (22) * | Age dependent | Days 1, (8), 15, (22) * |

# L-Asparaginase or PEG-asparaginase based on the drug availability.

Triple intrathecal treatments (methotrexate 8, 10 or 12 mg; hydrocortisone 16, 20 or 24 mg; and cytarabine 24, 30 or 36

mg for ages 1 to 1.99, 2 to 2.99 and ≥ 3 years, respectively)

*Triple intrathecal treatment was administered on days 8 and 15 for all patients; extra IT was administered on days 8, and 22 for patients with high-risk features of CNS relapse (CNS-2, CNS-3, traumatic lumbar puncture with blasts, and WBC > 50x109/L)

**B. FLAG chemotherapy**

|  | **Standard Therapy** | | **Augmented Therapy** | |
| --- | --- | --- | --- | --- |
| **Agent** | **Dose** | **Schedule** | **Dose** | **Schedule** |
| Fludarabine * | - | - | 15 mg/m2 or 30 mg/m2 | Days 2-5 |
| Cytarabine | - | - | 2 g/m2 | Days 2-5 |
| G-CSF | - | - | 5 mic/kg | Day 5 till ANC>1000 |
| Triple Intrathecal (IT) | - | - | Age dependent | Day 1 |

* Fludarabine was given as 15 mg/m2 in patients with residual leukemia cells in the bone marrow at end of induction >=0.01% and <1%, and at dose of 30 mg/m2 if residual leukemia cells>=1%.

**C. Consolidation Therapy**

|  | **Standard Therapy** | | **Augmented Therapy** | |
| --- | --- | --- | --- | --- |
| **Agent** | **Dose** | **Schedule** | **Dose** | **Schedule** |
| High-dose methotrexate | 5 g/m2 | Days 1, 15, 29 and 43 | 5 g/m2 | Days 1, 15, 29 and 43 |
| Mercaptopurine | 50 mg/m2/d | Days 1 to 56 | 50 mg/m2/d | Days 1 to 56 |
| Triple Intrathecal (IT) | Age dependent | Days 1, 15, 29 and 43 | Age dependent | Days 1, 15, 29 and 43 |

**D. Re-intensification Therapy**

|  | **Standard Therapy** | | **Augmented Therapy** | |
| --- | --- | --- | --- | --- |
| **Agent** | **Dose** | **Schedule** | **Dose** | **Schedule** |
| Dexamethasone | - | - | 20 mg/m2/d orally or IV | Days1-6 |
| Cytarabine | - | - | 2 g/m2 every 12 hours (4 doses) | Days 1-2 |
| Etoposide | - | - | 100 mg/m2 every 12 hours (5 doses) | Days 3-5 |
| L-Asparaginase or #  Peg-Asparaginase | - | - | 25,000 U/m2 IM or  2500 U/m2 IM | Day 6  Day 6 |
| Triple Intrathecal (IT) | - | - | Age dependent | Day 5 |

# L-Asparaginase or PEG-asparaginase based on the drug availability.

**E. Continuation Therapy**

| **Week** | **Standard Therapy** | **Augmented Therapy** |
| --- | --- | --- |
| 1 | Asparaginase# + mercaptopurine + dexamethasone  + vincristine + doxorubicin | Asparaginase#+ mercaptopurine + dexamethasone  + vincristine + doxorubicin |
| 2 | Asparaginase + mercaptopurine | Asparaginase + mercaptopurine |
| 3 | Asparaginase + mercaptopurine | Asparaginase + mercaptopurine |
| 4 | Asparaginase + mercaptopurine + dexamethasone  + vincristine + doxorubicin | Asparaginase + mercaptopurine + dexamethasone  + vincristine + doxorubicin |
| 5 | Asparaginase + mercaptopurine | Asparaginase + mercaptopurine |
| 6 | Asparaginase + mercaptopurine | Asparaginase + mercaptopurine |
| 7* | Reinduction I: Asparaginase + dexamethasone + vincristine + doxorubicin | Reinduction I: Asparaginase + dexamethasone + vincristine + doxorubicin |
| 8 | Reinduction I: Asparaginase + vincristine + doxorubicin | Reinduction I: Asparaginase + vincristine + doxorubicin |
| 9 | Reinduction I: Asparaginase + dexamethasone + vincristine | Reinduction I: Asparaginase + dexamethasone + vincristine |
| 10 | Asparaginase + mercaptopurine | Asparaginase + mercaptopurine |
| 11 | Asparaginase + mercaptopurine | Asparaginase + mercaptopurine |
| 12* | Asparaginase + mercaptopurine | Asparaginase + mercaptopurine |
| 13 | Asparaginase + mercaptopurine | Asparaginase + mercaptopurine |
| 14 | Asparaginase + mercaptopurine | Asparaginase + mercaptopurine |
| 15 | Asparaginase + mercaptopurine + dexamethasone  + vincristine + doxorubicin | Asparaginase + mercaptopurine + dexamethasone  + vincristine + doxorubicin |
| 16 | Asparaginase + mercaptopurine | Asparaginase + mercaptopurine |
| 17* | Reinduction II: Asparaginase + dexamethasone + vincristine | Asparaginase + mercaptopurine |
| 18 | Reinduction II: Asparaginase + vincristine | Asparaginase + mercaptopurine |
| 19 | Reinduction II: Asparaginase + dexamethasone + vincristine + high-dose cytarabine | Asparaginase + mercaptopurine |
| 20 | - | Mercaptopurine + methotrexate |
| 21 | Mercaptopurine + methotrexate | Mercaptopurine + methotrexate |
| 22 | Mercaptopurine + methotrexate | Mercaptopurine + methotrexate |
| 23 | Cyclophosphamide + cytarabine | Cyclophosphamide + cytarabine |
| 24 | Dexamethasone + vincristine | Dexamethasone + vincristine |
| 25 | Mercaptopurine + methotrexate | Mercaptopurine + methotrexate |
| 26* | Mercaptopurine + methotrexate | Mercaptopurine + methotrexate |
| 27 | Cyclophosphamide + cytarabine | Cyclophosphamide + cytarabine |
| 28 | Dexamethasone + vincristine | Dexamethasone + vincristine |
| 29 | Mercaptopurine + methotrexate | Mercaptopurine + methotrexate |
| 30 | Mercaptopurine + methotrexate | Mercaptopurine + methotrexate |
| 31* | Cyclophosphamide + cytarabine | Cyclophosphamide + cytarabine |
| 32 | Dexamethasone + vincristine | Dexamethasone + vincristine |
| 33 | Mercaptopurine + methotrexate | Mercaptopurine + methotrexate |

# L-Asparaginase or PEG-asparaginase based on the drug availability.

L-Asparaginase was administered at dose of 25,000 U/m2 weekly intramuscularly for 19 doses.

PEG-Asparaginase was administered at dose of 1000 U/m2 every two weeks intramuscularly for 10 doses.

Note: Reinduction II was omitted for patients treated per augmented therapy.

Mercaptopurine: 50 mg/m2 orally daily for 7 days between weeks 1 and 19 and 75 mg/m2 after week 1. Dexamethasone: 8 mg/m2 orally per day for 5 days; 8 mg/m2 on days 1 to 7 and days 15 to 21 during reinduction I (weeks 7 to 9) and reinduction II (weeks 17 to19). Methotrexate, 40 mg/m2 orally; doxorubicin, 30 mg/m2 intravenously; vincristine, 2 mg/m2 intravenously and

1.5 mg/m2 intravenously during reinductions I and II; cyclophosphamide, 300 mg/m2 intravenously; cytarabine, 300 mg/m2 intravenously. Patients received three drug pairs administered in 4-week blocks till week 32: mercaptopurine plus methotrexate in the first and second weeks, cyclophosphamide plus cytarabine in the third week, dexamethasone plus vincristine in the fourth week. After week 32, cyclophosphamide plus cytarabine continued every 8 weeks till week 66, and pulse of dexamethasone and vincristine continued every 12 weeks, with daily Mercaptopurine and weekly methotrexate till end of therapy. The total duration of continuation treatment was 120 weeks for girls and 146 weeks for boys.

* Triple Intrathecal therapy (IT) was administered on weeks 7, 12, 17, 26, 31, 35, 39, 43, 47, 51, 55, 59, 67, 75, 83, 91, 99.

**Table 2. Univariate Analysis using log-rank test according to clinical, biologic characteristic and treatment outcomes of whole T-cell ALL patients:**

| **Variables** | **P value EFS** | **P value**  **OS** |
| --- | --- | --- |
| Gender | 0.32 | 0.17 |
| CNS | 0.25 | 0.25 |
| **Standard vs Augmented** | **0.03** | **0.06** |
| Age (1-10 vs. others) | 0.35 | 0.22 |
| NCI risk categories | 0.058 | 0.074 |
| WBC>=50 | 0.69 | 0.81 |
| WBC>=100 | 0.59 | 0.88 |
| **BM at day 15_morphology** | **0.024** | **0.02** |
| MRD at end of Induction | 0.063 | 0.054 |
| Early Response | 0.135 | 0.11 |
| **Cranial XRT** | **0.00001** | **0.01** |

**Definitions:**

**Definitions of Bone Marrow Involvement:**

BONE MARROW STATUS:

M1: < 5% lymphoblasts

M2: 5 - 25% lymphoblasts

M3: > 25% lymphoblasts.

**Definitions of Early Response to Treatment**

Rapid Early Response (RER): M1 marrow on Day 15, and M1 marrow with negative MRD status (< 0.01%) at end of induction.

Slow Early Response (SER): M2 or M3 marrow on Day 15 OR positive MRD status (MRD>=0.01% at end of induction.
